# Astaxanthin Reduces Stemness Markers in BT20 and T47D Breast Cancer Stem Cells by Inhibiting Expression of Pontin and Mutant p53

**DOI:** 10.3390/md18110577

**Published:** 2020-11-20

**Authors:** Yong Tae Ahn, Min Sung Kim, Youn Sook Kim, Won Gun An

**Affiliations:** 1 Research Institute for Longevity and Well-Being, Pusan National University, Busan 46241, Korea; sytahn@pusan.ac.kr; 2 Division of Pharmacology, School of Korean Medicine, Pusan National University, Yangsan 50612, Korea; msk@pusan.ac.kr; 3 Gene & Cell Therapy Research Center for Vessel-Associated Diseases, Pusan National University, Yangsan 50612, Korea; younskim@pusan.ac.kr

**Keywords:** astaxanthin, T47D, BT20, pontin, mutp53, cancer stem cells, Oct4, Nanog, siRNA

## Abstract

Astaxanthin (AST) is a product made from marine organisms that has been used as an anti-cancer supplement. It reduces pontin expression and induces apoptosis in SKBR3, a breast cancer cell line. Using Western blotting and qRT-PCR analyses, this study revealed that in the T47D and BT20 breast cancer cell lines, AST inhibits expression of pontin and mutp53, as well as the Oct4 and Nanog cancer stem cell (CSC) stemness genes. In addition, we explored the mechanism by which AST eradicates breast cancer cells using pontin siRNAs. Pontin knockdown by pontin siRNA reduced proliferation, Oct4 and Nanog expression, colony and spheroid formation, and migration and invasion abilities in breast cancer cells. In addition, reductions in Oct4, Nanog, and mutp53 expression following rottlerin treatment confirmed the role of pontin in these cells. Therefore, pontin may play a central role in the regulation of CSC properties and in cell proliferation following AST treatment. Taken together, these findings demonstrate that AST can repress CSC stemness genes in breast cancer cells, which implies that AST therapy could be used to improve the efficacy of other anti-cancer therapies against breast cancer cells.

## 1. Introduction

Breast cancer, most frequently found in women with menopause, develops from breast tissue and is characterized by uncontrolled cell growth, with the ability to metastasize to other tissues [1]. Risk factors for breast cancer include environmental mechanisms, genetic elements, and lifestyle components [2]. Current synthetic chemical anti-cancer agents are restricted to use for the removal of breast cancer due to their cytotoxic effects on normal host cells [3]. Thus, natural therapeutic products that inhibit cancer cell growth without excessive toxicity are under investigation to identify candidate therapeutics [4].

Many marine organisms and algae have the red pigment astaxanthin (AST), a xanthophyll carotenoid (3,3′-dihydroxy-beta, beta-carotene-4,4′-dione) [5]. AST has some effects against various types of cancer (e.g., oral, bladder, colon, blood, liver, lung, and breast). These effects are mediated by anti-proliferative, pro-apoptotic, antioxidant, anti-inflammatory, anti-invasion and migration, and anti-gap junctional intracellular communication mechanisms [6]. Potential molecular targets for AST in cancers include NF-kB, STAT3, P13K/AKT, MAPKs, PPAR, Nrf2, and pontin [6,7].

Pontin is a conserved ATPase of the AAA+ superfamily, with Walker A and B motifs, arginine fingers, and insertion domains that are conserved from yeast to humans [8]. The pontin gene is located on chromosome 3q21 and encodes a 456-amino-acid protein [9]. In association with its partner, reptin, pontin forms a multi-protein complex that is presumably involved in activities such as DNA damage sensing and repair, cell viability and death, gene transcription regulation, telomerase activity, and chromatin remodeling, protein assembly, and ribonucleoprotein complexes [10]. Pontin has limited homology to the bacterial helicase RuvB, which is involved in the resolution of Holliday junctions during recombination [11]. Pontin and reptin are both overexpressed in many cancer types, but only pontin is overexpressed in breast cancer cells [10]. There is some evidence that pontin reduces ATPase activity, thereby contributing to apoptotic activity in cancer cells [12]. Pontin was recently found to interact with mutant p53 (mutp53) to promote a gain of function mutation [13]. Pontin also serves as a cofactor for octamer-binding transcription factor 4 (Oct4) during the maintenance of mouse embryonic stem cells (ESCs) [14].

The tumor suppressor protein p53 plays an important role in the suppression of human cancers [15]. However, its function is sometimes impaired because of frequent mutations [16]. Furthermore, mutp53 is restricted to poorly differentiated tumors, whereas the overexpression of wild-type p53 is restricted to less-differentiated areas of tumors [17]. These findings have demonstrated that mutp53 is associated with cancer stem cell (CSC) formation and poor prognosis [18]. Recently, a correlation between mutp53 and CSC phenotype has been reported [19]. CSCs, also known as tumor-initiating cells, are a subpopulation of tumor cells that can generate heterogeneous progenies [20]. The proportion of CSCs in an overall cancer cell population is approximately 0.05–3%, but these cells are considered to be the fundamental barrier to operative cancer therapy due to their distinct cellular properties (e.g., self-renewal, differentiation, and tumor formation) [21]. CSCs are commonly identified in breast cancer using specific biomarkers including CD44, CD133, CD166, CD24, CD29, EpCAM, and aldehyde dehydrogenase 1 [22]. In addition, there is increasing evidence that mutp53 is involved in the expression of Oct4 and Nanog, which are crucial components for maintaining the pluripotency and self-renewal properties of normal stem cells and ESCs [14,23]. These genes promote each other’s expression, control cancer progression, and are biomarkers of CSCs [24]. Oct4 belongs to the Pit-Oct-Unc (POU) family of transcriptional factors and plays a crucial role in stem cell differentiation and pluripotency by determining the fate of ESCs [25]. It also contributes to tumorigenesis and self-renewal through activation of its downstream target genes (e.g., Nanog, Sox2, and Klf4) [25]. Nanog is a homeobox domain transcription factor expressed in human cancers [26]. It plays a key role in malignant disease through effects on cell proliferation and traits such as clonogenic growth, tumorigenicity, invasiveness, and therapeutic resistance [26].

Previously, we reported that AST modulates pontin expression to cause apoptosis in breast cancer cells [7]. In this study, we explored how AST-targeted pontin functions in the obliteration of breast cancer cells and concluded that AST might impede breast cancer growth by reducing the populations of CSCs. These data demonstrate that the marine compound AST is a powerful anticancer agent.

## 2. Results

### 2.1. AST Reduces the Expression Levels of Pontin, mutp53, Oct4, and Nanog in T47D and BT20 Breast Cancer Cells, Inhibiting Their Proliferation

**Figure 1:**
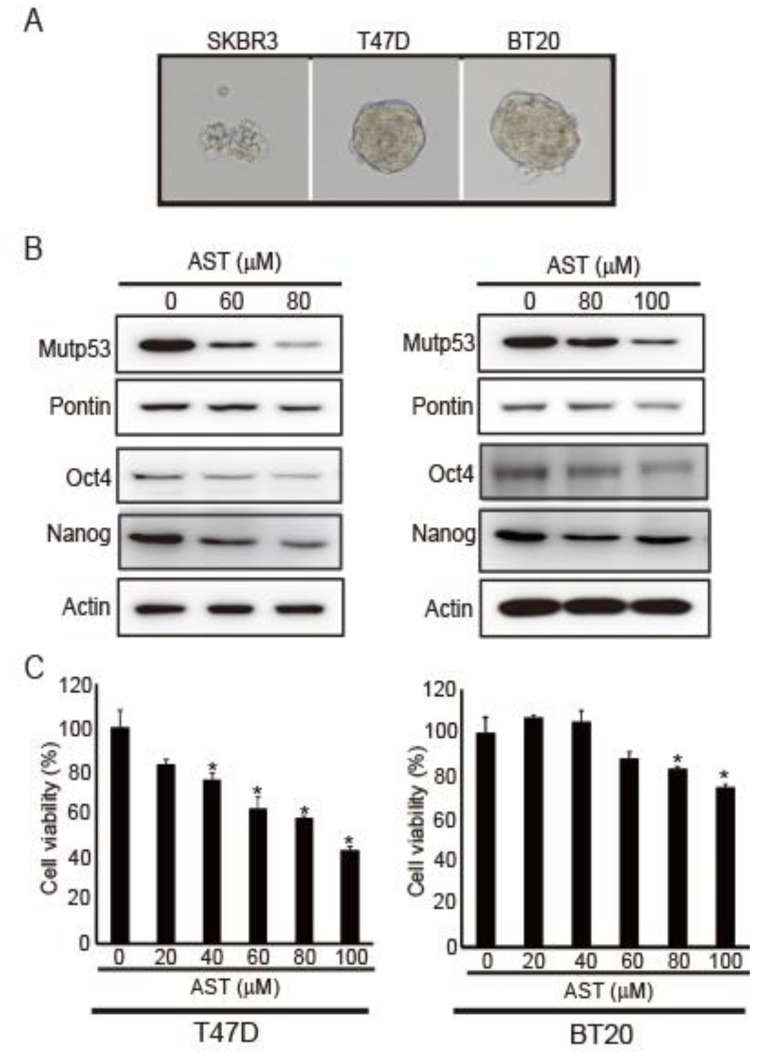
AST reduced the expression levels of pontin, mutp53, Oct4, and Nanog in T47D and BT20 breast cancer cell lines, thereby inhibiting cell proliferation. (**A**) Spheroid formation abilities of SKBR3, T47D, and BT20 breast cancer cell lines. (**B**) Expression levels of mutp53, pontin, Oct4, and Nanog were determined via Western blotting of T47D and BT20 cells after AST treatment. Actin was used as a loading control. (**C**) Cells were incubated for 48 h at 37 °C at the indicated concentrations of AST in a 96-well plate. Then, CCK-8 was added and the cells were incubated for 3 h at 37 °C. Absorbance was measured with a spectrophotometer at 450 nm. Data are expressed as the mean ± standard deviation (SD) of three independent experiments. * *p* < 0.05.

AST reduces pontin expression and mutp53 in the SKBR3 breast cancer cell line [7]. Moreover, the interaction between pontin and mutp53 promotes cancer migration and cancer stemness progression [13]. Thus, we investigated whether AST could affect cancer stemness properties in breast cancer cells. Because spheroid formation ability is a characteristic of CSCs [27], we investigated spheroid formation ability in SKBR3, T47D, and BT20 breast cancer cell lines. T47D and BT20 cells showed spheroid formation, whereas SKBR3 cells did not (Figure 1A). Because SKBR3 cells failed to form spheroids, we performed subsequent experiments with T47D and BT20 cells.

First, to determine whether AST could regulate the expression levels of pontin, mutp53, Oct4, and Nanog in T47D and BT20 cells, their levels were measured via Western blotting. As the concentration of AST increased, the expression levels of pontin, mutp53, Oct4, and Nanog decreased relative to control (Figure 1B). These findings indicate that AST can regulate CSC genes in both T47D and BT20 cells.

Next, cell viability was measured using a CCK-8 viability assay to determine whether AST would affect the growth of T47D and BT20 cells. Cell growth was clearly inhibited by AST treatment in a dose-dependent manner (Figure 1C). Therefore, AST can inhibit the proliferation of both T47D and BT20 breast cancer cell lines.

### 2.2. Pontin Knockdown Attenuates the Proliferation of T47D and BT20 Breast Cancer Cells

**Figure 2:**
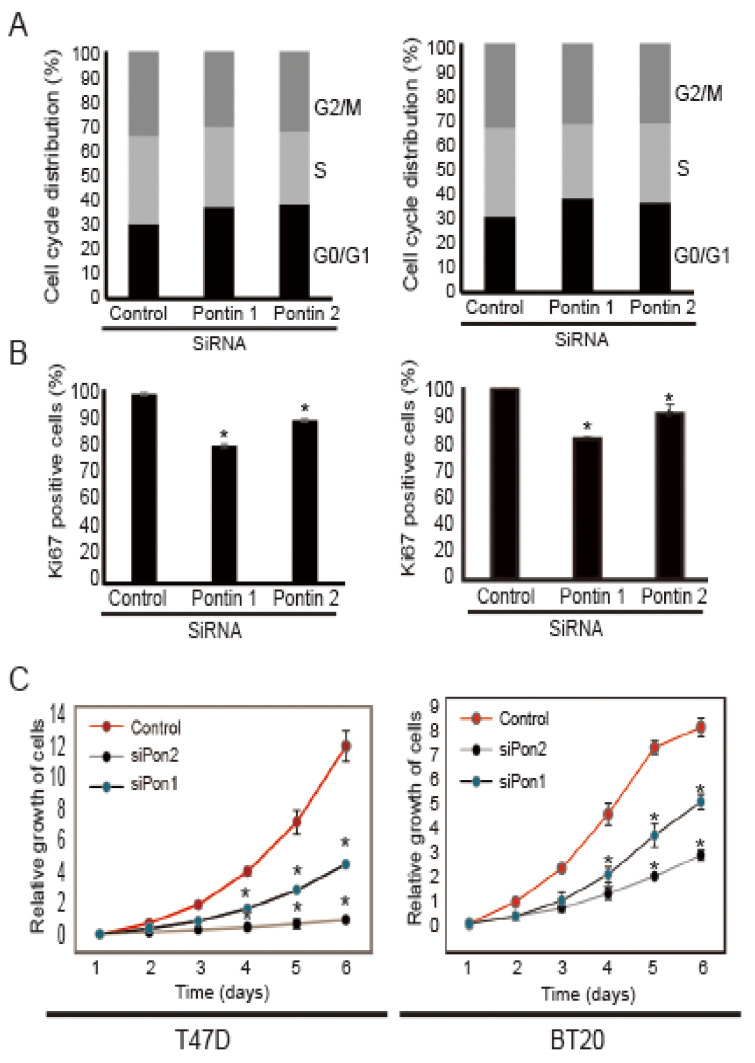
Pontin knockdown attenuated the proliferation of T47D and BT20 cells. (**A**) Cell cycle analyses of T47D and BT20 cells after targeted pontin knockdown. Cells were harvested 3 days after transfection of pontin siRNAs or control siRNA. Similar results were obtained from three independent experiments. (**B**) Ki67 incorporation was used to determine the proportions of cells in each cell cycle phase. Cells were harvested 3 days after transfection of control siRNA or pontin siRNAs. Proportions of Ki67-positive cells are shown. Results are expressed as the mean ± SD of three independent experiments. * *p* < 0.05. (**C**) Growth curves of T47D and BT20 cells after targeted pontin knockdown. Data are represented the mean ± SD of three independent experiments (* *p* < 0.05).

To determine whether pontin is necessary for the proliferation of breast cancer cells, we performed cell cycle arrest, Ki67 staining, and CCK8 viability assays following the induction of pontin silencing. As shown in Figure 2A, the cell cycle profiles of T47D cells treated with pontin siRNAs included significantly greater proportions of cells in the G0/G1 phase (siPontin1: 39.06% ± 0.57%, siPontin2: 41.07% ± 1.72%), compared to the control siRNA group (29.1% ± 0.7%). This trend was also evident in BT20 cells treated with pontin siRNAs (siPontin1; 37.06% ± 0.57%, siPontin2; 36.07% ± 1.72%), compared to the control siRNA group (28.0% ± 0.7%). Conversely, the proportions of cells in the G2/M phase after pontin siRNA transfection were significantly reduced in both cell lines. Therefore, pontin is a key molecule for the proliferation of breast cancer cells.

A Ki67 incorporation experiment showed reductions in the number of Ki67-positive cells following pontin siRNA treatment, compared to control siRNA (Figure 2B), indicating that pontin is an important molecule for cell proliferation. To examine whether downregulation of pontin would affect cancer cell growth, CCK8 viability assays were conducted (Figure 2C). The numbers of cells were significantly lower in pontin siRNA groups than in control siRNA groups after transfection.

Taken together, these data indicate that pontin depletion leads to defects in breast cancer cells, which implies that it plays a crucial role in the proliferation of breast cancer cells.

### 2.3. Pontin Knockdown Reduces the Levels of mutp53, Oct4, and Nanog in T47D and BT20 Breast Cancer Cells

**Figure 3:**
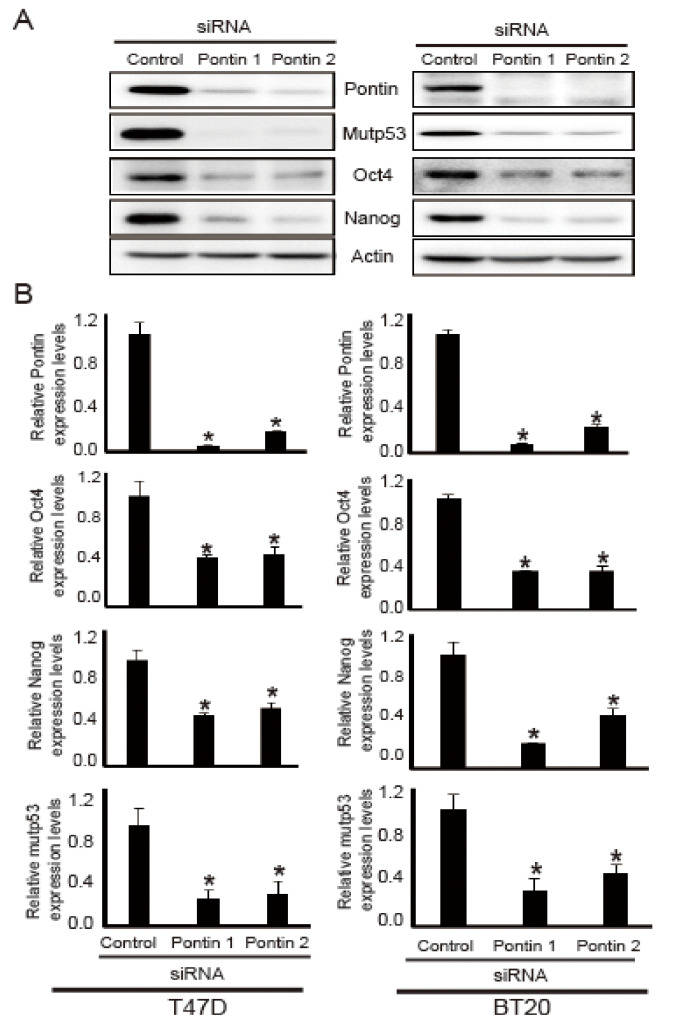
Effects of pontin-targeting siRNAs on expression levels of Oct4, Nanog, and mutp53 in T47D and BT20 breast cancer cell lines. Cells were transfected with pontin-targeting or control siRNA. (**A**) Cell lysates were prepared from T47D and BT20 cells, and the expression levels of Oct4, Nanog, and mutp53 were detected by Western blotting. (**B**) Total RNA was extracted, and the expression levels of Oct4, Nanog, and mutp53 were evaluated via qRT-PCR. All experiments were normalized by comparison with glyceraldehyde 3-phosphate dehydrogenase (GAPDH). Data represent the mean ± SD of three independent experiments (* *p* < 0.05).

Because AST reduced the expression levels of pontin, mutp53, Nanog, and Oct4, their levels were analyzed by Western blotting and quantitative reverse transcriptase polymerase chain reaction (qRT-PCR) analyses following pontin knockdown in T47D and BT20 cells. As shown in Figure 3A, after transfection of pontin siRNAs, the protein expression levels of mutp53, Nanog, and Oct4 were downregulated compared to control.

To confirm the Western blotting results, the mRNA expression levels of pontin, mutp53, Nanog, and OCT4 in both T47D and BT20 cells were investigated via qRT-PCR. As shown in Figure 3B, pontin siRNA groups had reduced mRNA expression levels of pontin, mutp53, Nanog, and OCT4 in both T47D and BT20 cells. These results suggest that pontin knockdown in T47D and BT20 cells can reduce the expression levels of mutp53, Nanog, and Oct4. Thus, pontin could be a crucial component of cancer stemness properties in T47D and BT20 breast cancer cell lines.

### 2.4. Pontin Knockdown Reduces Colony and Spheroid Formation Ability in T47D and BT20 Breast Cancer Cells

Pontin is a necessary cofactor for Oct4 during the maintenance of mouse ESCs [14]. Thus, pontin might regulate colony and spheroid formation in breast cancer cells because these abilities are essential CSC characteristics [27].

**Figure 4:**
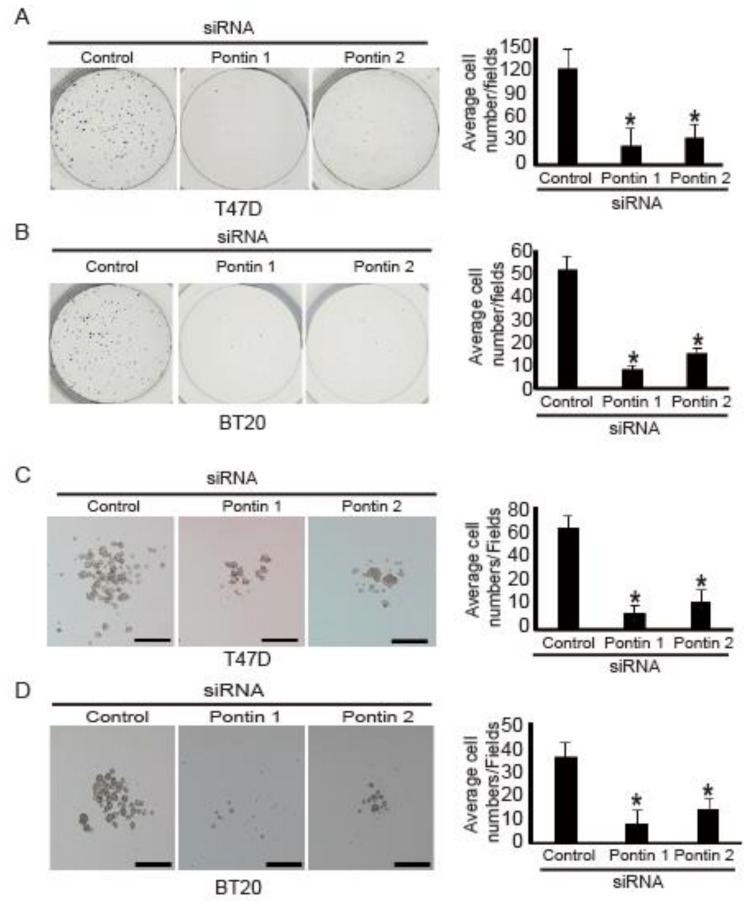
Pontin knockdown reduced colony and spheroid formation abilities in T47D and BT20 cells. Pontin knockdown with siRNA attenuated colony formation by (**A**) T47D and (**B**) BT20 cells. Left panel, representative images from a portion of field; right panel, quantification of average number of migrated cells/field. Pontin knockdown with siRNA attenuated spheroid formation by (**C**) T47D and (**D**) BT20 cells. Left panel, representative images from a portion of field; right panel, quantification of average number of migrated cells/field. Data represent the mean ± SD of three independent experiments (* *p* < 0.05).

To test this hypothesis, colony formation assays were performed using T47D and BT20 cells transfected with pontin siRNAs. After 14 days in culture, the numbers of colonies formed by cells transfected with pontin siRNA were substantially reduced, compared to the numbers of T47D cells (Figure 4A) and BT20 cells (Figure 4B) transfected with control siRNA.

Next, because CSCs have been shown to form floating spheres in culture [27], their abilities to form spheroids in vitro were measured after pontin knockdown. Control siRNA or pontin siRNAs were transfected into the cells, which then were grown in suspension with serum-free spheroid media for spheroid maintenance. After 7 days in culture, the numbers of spheroids formed in cells transfected with pontin siRNAs were considerably reduced, compared to the numbers of T47D cells (Figure 4C) and BT20 cells (Figure 4D) transfected with control siRNA.

Taken together, these results indicate that pontin knockdown in breast cancer cells might attenuate the properties of CSCs.

### 2.5. Pontin Knockdown Reduces Migration and Invasion Abilities in T47D and BT20 Breast Cancer Cells

**Figure 5:**
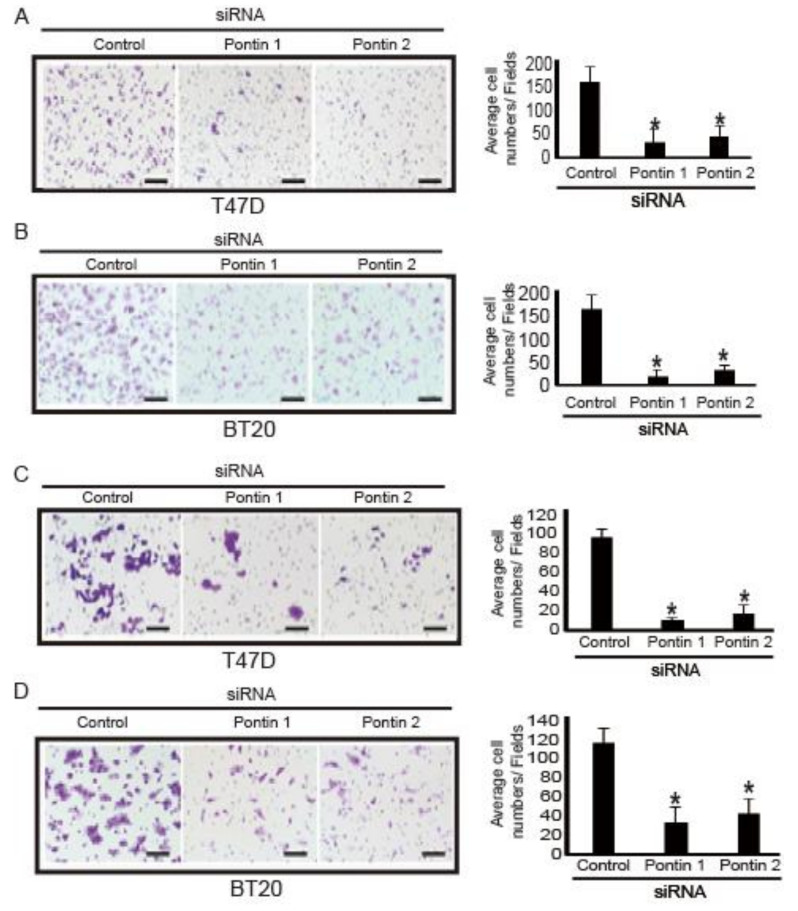
Pontin knockdown reduced migration and invasion in breast cancer cells. Pontin knockdown by siRNA attenuated migration of (**A**) T47D and (**B**) BT20 cells, as determined by transwell migration assays. Left panel, representative images from a portion of field; right panel, quantification of average number of migrated cells/field (×200 magnification). Pontin knockdown by siRNA attenuated invasion of (**C**) T47D and (**D**) BT20 cells, determined using Matrigel-coated transwell invasion assays. Left panel, representative images from a portion of field; right panel, quantification of average number of migrated cells/field (×200 magnification). Data represent the mean ± SD of three independent experiments (* *p* < 0.05).

Because specific depletion of pontin was able to change colony and spheroid formation abilities, other CSC abilities (e.g., migration and invasion) were presumably changed. To test this hypothesis, transwell migration and invasion assays were performed using cells transfected with pontin siRNAs or control siRNA. As shown in Figure 5, migration and invasion of breast cancer cells were inhibited by pontin knockdown, compared to controls. The migration inhibition rates of T47D and BT20 cells were 82% and 85%, respectively. The invasion inhibition rates of T47D and BT20 cells were 78% and 64%, respectively. These results suggest that pontin knockdown in breast cancer cells attenuates the properties of CSCs.

### 2.6. Rottlerin Reduces Expression Levels of Oct4, Nanog, and mutp53 in T47D and BT20 Breast Cancer Cells

**Figure 6:**
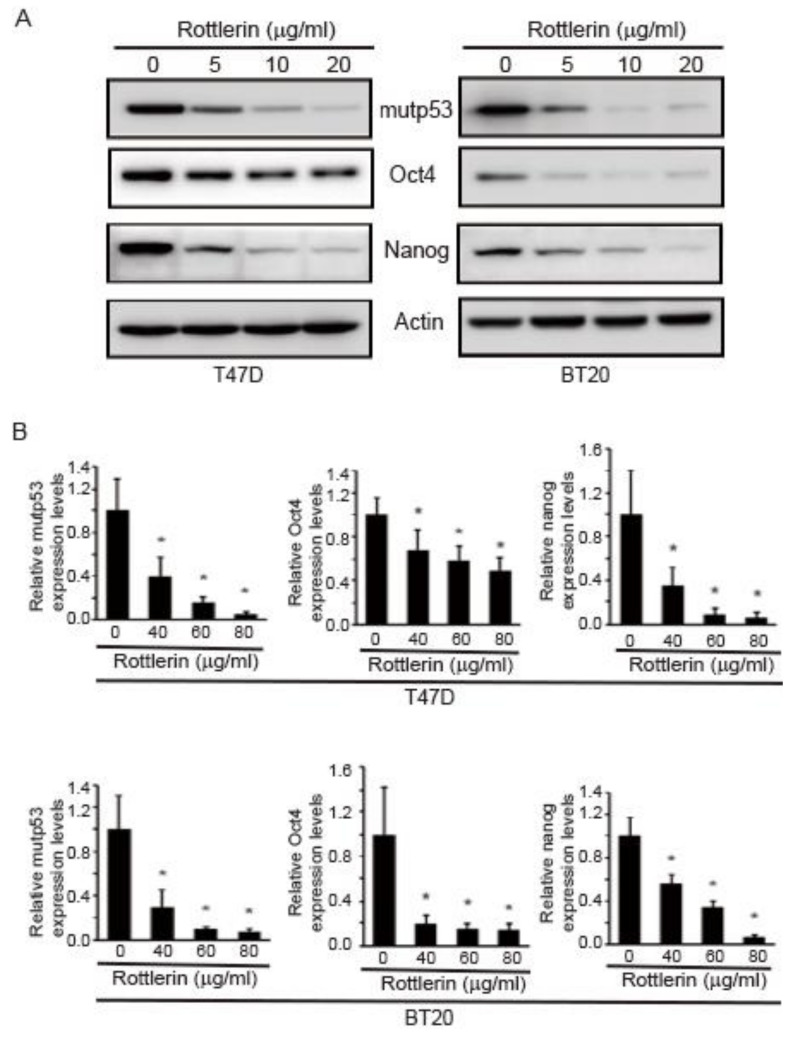
Rottlerin reduced expression levels of Oct4, Nanog, and mutp53 in T47D and BT20 breast cancer cell lines. Cell lysates were prepared from T47D and BT20 cells that had been subjected to rottlerin treatment. Expression levels of Oct4, Nanog, and mutp53 were detected via Western blotting analyses and qRT-PCR. (**A**) Cell lysates were prepared from T47D and BT20 cells, and the expression levels of Oct4, Nanog, and mutp53 were detected by Western blotting. (**B**) Total RNA was extracted, and the expression levels of Oct4, Nanog, and mutp53 were evaluated by qRT-PCR. All expression levels were normalized to GAPDH. Data represent the mean ± SD of three independent experiments (* *p* < 0.05).

To further demonstrate the role of pontin in this study, rottlerin (a pontin-specific ATPase inhibitor) was used. The expression levels of mutp53, Nanog, and Oct4 under rottlerin treatment were measured via Western blotting and qRT-PCR analyses. As shown in Figure 6A, rottlerin diminished the expression levels of mutp53, Oct4, and Nanog in T47D and BT20 cells.

To confirm these findings, the mRNA expression levels of mutp53, Nanog, and Oct4 in rottlerin-treated T47D and BT20 cells were investigated via qRT-PCR. With increasing rottlerin concentration, the mRNA expression levels of mutp53, Nanog, and Oct4 decreased in both T47D and BT20 cells (Figure 6B). These results confirm that pontin regulates the expression levels of mutp53, Nanog, and Oct4 in breast cancer cells.

## 3. Discussion

AST obtained from marine organisms has powerful antioxidant, anti-inflammatory, and anti-cancer properties. In the present study, the anti-cancer effects of AST on breast cancer cells were investigated. Because AST regulates pontin expression and causes apoptosis in SKBR3 cells [7], its effects were further explored to identify the molecules that it regulates, as well as how pontin impedes breast cancer cell growth, using a pontin knockdown approach.

Pontin is an AAA+ ATPase that possesses both ATPase and DNA helicase activities [8,11]. Its overexpression has been observed in many cancers [28]. Its ATPase activity is important for cell proliferation in many tumors, including breast cancer and hepatocellular carcinoma [10]. In this study, pontin was found to participate in the proliferation of the T47D and BT20 breast cancer cell lines. Pontin is closely associated with various cell cycle-related genes, including E2F1 and RB [29]. The downregulation of pontin leads to cell cycle arrest, resulting in the accumulation of cells in the G1 phase and the reduction of cells in the S and G2/M phases [30]. In this study, pontin knockdown in T47 and BT20 cells led to a significant reduction in cell growth (Figure 2A). In addition, cell cycle analyses revealed that pontin siRNA induced G1 cell cycle arrest, indicating that pontin is a crucial component in breast cancer cell proliferation.

Pontin negatively regulates mutant p53 during cancer development [13]. It can also cause a gain of function in mutp53, supporting tumorigenesis [13]. Its interaction with mutp53 promotes cancer stemness properties such as proliferation, metastasis, and invasion [18]. A critical role for mutp53 in CSC production is supported by the association between tumors with mutp53 and the undifferentiated phenotypes in these tumors [17]. Moreover, mutp53 has been shown to encourage a stem-like phenotype in breast cancers [31], while undifferentiated breast tumors express specific stemness genes [32,33]. Therefore, pontin was presumed to regulate specific stemness genes and markers by mediating mutp53 activity. Pontin and mutp53 are considered important regulatory components within the CSC circuitry. Additional studies are needed to determine whether pontin can regulate the expression of Oct4 and Nanog through a mutp53-related mechanism and to determine how it selectively regulates the expression of mutp53 and stemness genes in cancer cells.

CSCs have unique stemness properties concerning self-renewal, differentiation, and proliferative capacities. Notably, they exhibit robust expression of stemness genes including Oct4 and Nanog [34]. In CSCs, the expression of Nanog and Oct4 is associated with a more aggressive tumor phenotype [35]. Therefore, CSCs are presumed to promote the growth and development of most human malignancies. Nanog is expressed in cancer cells, where it enhances tumorigenesis by activating CSCs [36]. Moreover, Nanog and mutp53 are co-expressed in cancer cells [37]. Oct4 plays a key role in stem cell differentiation and pluripotency by determining the fate of ESCs [25]. Notably, pontin depletion causes downregulation of Oct4 in ESCs [14]. Pontin serves as a co-activator for Oct4-dependent long intergenic non-coding RNA transcription [14], indicating that it regulates Oct4 and Nanog expression. Here, we confirmed that AST repressed Oct4 and Nanog expression in T47D and BT20 cells.

Drug resistance is a well-known characteristic of CSCs [38]. CSCs possess high aldehyde dehydrogenase 1 activity and express CD44 but not CD24 [39,40,41]. Aldehyde dehydrogenase 1 overexpression is associated with poor prognosis and cancer recurrence after chemotherapy [42]. Furthermore, mutp53-dependent drug resistance is mediated by enhanced CSC properties [42]. CSCs can undergo cell cycle arrest, thereby resisting chemotherapy and radiotherapy [43]. Notably, tumor invasion and recurrence after radiotherapy and chemotherapy have been attributed to CSCs [44]. Therefore, therapeutic targeting of CSCs may improve the prognosis of patients with cancer [45]. Our findings imply that AST might be useful for the treatment of breast cancer because of its ability to repress CSC genes.

In conclusion, AST is a robust antioxidant that blocks the proliferation of T47D and BT20 breast cancer cells. It also inhibits the expression of pontin, mutp53, Oct4, and Nanog, which constitute CSC stemness genes in T47D and BT20 cells. In addition, the use of pontin siRNA in this study revealed the mechanisms by which AST eradicates breast cancer cells. Pontin knockdown reduced cell proliferation, Oct4 and Nanog expression, colony and spheroid formation, and migration and invasion in both T47D and BT20 cells. In addition, the reduction of Oct4, Nanog, and mutp53 expression levels following rottlerin treatment confirmed the role of pontin in these cells. Therefore, pontin might play a central role in the regulation of CSC properties and the proliferation of cancer cells, following addition of AST. Taken together, our findings suggest that AST could eradicate CSCs and serve as a powerful treatment for breast cancer. Further studies are needed to elucidate how AST represses the expression of CSC genes at the transcriptional or translational levels, what quantities of AST provide optimal cancer treatment effects, and the relationship between pontin and hsp90 during AST treatment for breast cancer.

## 4. Materials and Methods

### 4.1. Chemicals and Antibodies

Dulbecco’s modified Eagle medium (DMEM), fetal bovine serum (FBS), and Dulbecco’s phosphate-buffered saline (DPBS) were purchased from Gibco (Grand Island, NY, USA). Monoclonal antibodies specific for pontin, Oct4, Nanog, p53, and actin were obtained from Cell Signaling Technology (Danvers, MA, USA).

### 4.2. Cell Culture and Transfection

Human breast cancer cell lines SKBR3, T47D, and BT20 (American Type Culture Collection, Manassas, VA, USA) were cultured in DMEM containing 10% (*v*/*v*) FBS and 1% (*v*/*v*) penicillin-streptomycin (Gibco) at 37 °C in a 5% CO_2_ incubator. Cells were subcultured by enzymatic digestion with trypsin– ethylenediaminetetraacetic acid (EDTA) (0.25% trypsin and 1 mM EDTA) solution when they reached approximately 80% confluency. For siRNA targeting knockdown, siRNA oligos against pontin were purchased from Integrated Bioneer Corporation (Daejeon, Korea). Pontin-targeting siRNA sequences were as follows: siRNA-1, 5′-CAUGGGAGGAUAUGGCAAA-3′; siRNA-2, 5′-GAGGAUAUGGCAAAACCAU-3′. The control siRNA sequence was as follows: 5′-CCUACGCCACCAAUUUCGU-3′. The Neon Transfection System (Invitrogen) was used for electroporation to transfect T47D and BT20 cells with pontin siRNAs or control siRNA. Briefly, 2 × 10^6^ cells in buffer R were electroporated in 100 µL tips with 500 ng of siRNA, then plated with culture media in a 100 mm culture plate. The electroporation parameters were cell-line-specific (T47D: 1400 V, 20 mA, two pulses; BT20:1300 V, 20 mA, one pulse). For rottlerin treatment, cells were treated with various concentrations of rottlerin (Calbiochem, Billerica, MA, USA) for 12 h.

### 4.3. Cell Proliferation Assay

Cell proliferation was determined using a CCK-8 assay (Dojindo, Tokyo, Japan). For growth rate analyses, control and pontin knockdown cells were seeded with culture medium in 96-well plates (SPL Life Sciences, Seoul, Korea) at a density of 5 × 10^3^ cells per well. After incubation for 24 h at 37 °C, CCK-8 in culture medium was added to each well and cells were incubated for 2 h at 37 °C. The optical density was read at 450 nm using an ELISA reader (Tecan Group Ltd., Männedorf, Switzerland). Results were expressed as the mean ± SD of three independent experiments.

### 4.4. Cell Cycle Analyses

For cell cycle analyses, the Muse^®^ Cell Cycle Assay Kit (Millipore, Burlington, MA, USA) was used to determine the proportions of cells in each cell cycle phase. In brief, cells were transfected with pontin siRNAs or control siRNA, then harvested and fixed in 70% ethanol at −20 °C for 12 h. After fixation, the cells were washed twice with DPBS and stained with cell cycle solution for 20 min at room temperature in the dark. Then, the cells were counted using a Muse Cell Analyzer (Millipore). In addition, the Muse^®^ Ki67 Proliferation Kit (Millipore) was used to measure the populations of proliferating cells. Briefly, harvested cells were washed three times in ice-cold DPBS and suspended in fixation solution for 15 min. Then, they were transferred to permeabilization solution and incubated for 15 min. Next, they were washed three times in ice-cold DPBS and suspended in assay buffer with the hKi67-PE antibody for 15 min. Finally, they were counted using a Muse Cell Analyzer.

### 4.5. Western Blotting Analyses

Cells were collected by centrifugation at 4 °C, washed three times in ice-cold DPBS, and suspended in Radioimmunoprecipitation assay (RIPA) lysis buffer (50 mM Tris (pH 7.5), 2 mM EDTA, 100 mM NaCl, 1% NP-40) containing protease inhibitor cocktail (Sigma-Aldrich, St. Louis, MO, USA). Then, cell lysates were centrifuged at 13,000 rpm for 15 min at 4 °C, and the protein concentrations were determined using a bicinchoninic acid protein assay kit (Thermo Fisher Scientific, Rockford, IL, USA). Equal amounts of total protein (30 µg) were resolved by 8–12% SDS–PAGE and proteins were transferred to polyvinylidene difluoride (PVDF) membranes (Millipore). The membranes were blocked with 5% (*v*/*v*) skim milk in Tris-buffered saline with Tween (TBS-T; 50 mM Tris, 150 mM NaCl, and 0.1% Tween-20), incubated with specific primary antibodies in blocking buffer overnight at 4 °C, washed three times in TBS-T, and incubated for 1 h at room temperature with horseradish peroxidase-conjugated secondary antibody. Immunoreactive bands were developed using an enhanced chemiluminescence detection system (ECL Plus, Thermo Fisher Scientific).

### 4.6. Tumor Spheroid Formation Assay

T47D and BT20 cells were transfected with siRNA oligos targeting pontin or control for 24 h. Harvested cells from SKBR3, T47D, and BT20 cells or transfected T47D and BT20 cells were suspended in 24-well ultra-low attachment surface plates at 400 cells per well in spheroid media (serum-free DMEM/F12 media supplemented with 20 ng/mL hEGF, 10 ng/mL β-FGF, 2% B-27 supplement, and penicillin-streptomycin solution). Following 2 weeks of cultivation, spheroid formation was assessed using an Olympus IX51 microscope (Olympus, Waltham, MA, USA). Results were recorded for at least three independent experiments.

### 4.7. Cell Invasion Assay

Transwell chambers were used to examine cell invasion capability. T47D and BT20 cells were transfected with pontin-targeting or control siRNA oligos for 48 h. For transwell assays, the transwell chambers were coated with 100 µL BD Biosciences Matrigel™ overnight in a cell incubator. Then, transfected cells were trypsinized and resuspended at 1 × 10^5^ cells in 200 µL serum-free medium. The resuspended cells were placed into the upper chambers of a 24-well transwell plate (8 mm pore size; Corning Inc., Corning, NY, USA). The lower chambers were filled with 700 µL complete medium with 10% FBS. Following incubation for 24 h at 37 °C, non-invading cells were removed from the upper chamber with a cotton swab. Invading cells on the lower surface of the inserts were fixed with 4% paraformaldehyde and stained with 1% crystal violet. Three random fields for each insert were counted at 200× magnification. Data were recorded as the mean ± SD of at least three independent experiments.

### 4.8. Cell Migration Assay

The migration assay was performed using transwell chambers. T47D and BT20 cells were transfected with pontin-targeting or control siRNA oligos for 48 h. Then, transfected cells were trypsinized and resuspended at 2 × 10^4^ cells in 200 µL serum-free medium. The resuspended cells were placed into the upper chambers of a 24-well transwell plate (8 mm pore size; Corning Inc.). The lower chambers were filled with 700 µL complete medium with 10% FBS. Following incubation for 24 h at 37 °C, non-invading cells were removed from the upper chamber with a cotton swab. Invading cells on the lower surface of the inserts were fixed with 4% paraformaldehyde and stained with 1% crystal violet. Three random fields for each insert were counted at 200× magnification. Data were recorded as the mean ± SD of at least three independent experiments.

### 4.9. RNA Isolation and qRT-PCR

Total RNA was extracted using an RNeasy mini kit (Qiagen, Hilden, Germany) from T47D and BT20 cells transfected with pontin-targeting or control siRNA oligos, in accordance with the manufacturer’s instructions. Total RNA was reverse-transcribed into cDNA using Superscript II Reverse Transcriptase (Invitrogen, Carlsbad, CA, USA), in accordance with the manufacturer’s instructions. The cDNA was amplified by qPCR in an ABI 7500 real-time PCR system (Applied Biosystems, Foster City, CA, USA) using the SYBR Select Master Mix kit (Applied Biosystems) and with gene-specific primers. The thermocycling conditions were initial denaturation at 95 °C for 15 min, followed by 30 cycles of 95 °C for 20 s, 58 °C for 40 s, and 72 °C for 1 min. The results were normalized by comparison with the housekeeping gene GAPDH. The primer sequences used in this study were as follows: pontin forward, 5′-TGA AGA GCA CTA CGA AGA CG-3′; pontin reverse, 5′-AAC AAG ACA GCT CTT CCA GC-3; mutp53 forward, 5′-AGA AAA CCT ACC AGG GCA GC-3′; mutp53 reverse, 5′-CTC CGT CAT GTG CTG TGA CT-3′; Oct4 forward, 5′-CGA CTA TGC ACA ACG AGA GG-3′; Oct4 reverse, 5′-AGA GTG GTG ACG GAG ACA G-3′; Nanog forward, 5′-TCT TCC TAC CAC CAG GGA TG-3′; Nanog reverse, 5′-ATG CAG GAC TGC AGA GAT TC-3′; GAPDH forward 5′-ATA TGA TTC CAC CCA TGG CAA-3′; and GAPDH reverse, 5′-GAT GAT GAC CCT TTT GGC TCC-3. The expressions of genes including Pontin, mutp53, Oct4, and nanog were quantified using the ΔΔCt method.

### 4.10. Statistical Analyses

Statistical analyses were performed using SPSS Statistics Ver. 23 (IBM Corp., Armonk, NY, USA). Results are presented as the mean ± SD. Comparisons were performed using independent *t*-tests. *p* values < 0.05 were considered statistically significant.
